# School dietary habits & oral health experiences of primary school children in Johannesburg

**DOI:** 10.1371/journal.pone.0323522

**Published:** 2025-05-28

**Authors:** Octavia Refiloe Lebele, Veerasamy Yengopal, Peedi Mathobela, Mpho Matlakale Molete

**Affiliations:** 1 University of KwaZulu Natal, KZN Province, Durban, South Africa; 2 Faculty of Dentistry Western Cape Province, University of the Western Cape, Cape Town, South Africa; 3 Department of Internal Medicine, University of the Witwatersrand, Johannesburg, Gauteng, South Africa; 4 Department of Oral Biological Sciences, University of the Witwatersrand, Johannesburg, Gauteng, South Africa; King Faisal University, SAUDI ARABIA

## Abstract

**Background & aim:**

Understanding the impact of food exposures outside the home environment is pivotal for helping children establish healthy eating patterns, for the prevention and delay of dental caries and other non-communicable diseases. This study sought to assesses the dietary habits and oral health experiences of primary school children in Johannesburg.

**Methods:**

This was a cross-sectional analytical study of grade three learners, aged 9–11 years old. The measurement tools used included an observational checklist, a questionnaire, an oral health examination and anthropometric measures for assessing the Body Mass Index (BMI). Correlation and regression analysis were undertaken to determine relationships between sugar intake, calories intake, dmft, DMFT and gingival index.

**Results:**

Of the 107 eligible children participating, 68% were from a school with a feeding scheme and 31.8% with no feeding scheme. The mean (SD) of BMI, dmft, and DMFT were 18.19 (3.59), 3.14 (3.39), and 1.49 (2.10), respectively. The mean sugar content of meals served in schools with a feeding scheme was lower [11.65g (SD 9.6)] than the mean sugar content of food consumed at a schools with no feeding scheme [35.84g (29.9)]. The regression analysis in this population indicated that the low gingival score was associated with high BMI and sugar intake.

**Conclusion:**

Although the sugar content of meals served at National School Nutrition Programme (NSNP) schools was less that the sugar content accessed by children at Non-NSNP schools, both the schools had poor controls over sugar consumption and purchasing behaviour of the learners. The low gingival score was attributed to socio-economic status and access to toothbrushes and pastes.

## Introduction & background

Dental caries remains one of the most common conditions among children even though studies have shown some reduction in its prevalence over the past 20 years due to improved education, dental hygiene, sealants, and water fluoridation [1]. The significant link between sugar intake, poor diet and dental caries has been shown over the years to have become a persistent challenge [2]. Globally, children who suffer from poor oral health are more likely to have restricted activity days as a result of pain caused by toothache or dental infection [3]. Furthermore, over 50 million school hours are lost annually because of oral health problems, which affect children’s performance at school [4].

The published literature has shown that school oral health programs can provide an effective platform for preventing dental caries and promoting oral health because they reach over 350 million of children worldwide per day [5]. A large part of the children’s waking hours are spent at school. Additionally, the school food environment can have a significant impact on learners’ health and oral health and it is often the place where lifelong nutritional habits are formed [6].

In response to high levels of malnutrition in the country, the South African government introduced the Primary School Nutrition Programme (PSNP) under the guidance of the Department of Health and Education to promote and advocate for malnutrition control, nutritional education, and healthy dietary habits among children from low socioeconomic households. Malnutrition refers to both under nutrition and over nutrition, ranging from severe nutrient deficiencies to extreme obesity [7,8]. The program was renamed the National School Nutrition Program (NSNP), and implementation was transferred to the Department of Education in 2004 to strengthen implementation and support [9]. The NSNP is regulated by the government in alignment with regulations provided by the WHO, World Bank, and the World Food Programme (WFP) [8]. Whilst these foods served within the NSNP are considered healthy and nutritious, competitive foods that learners have exposure to are not regulated and these include food from lunch boxes, vending machines snacks, fast food stalls, and school vendors [10]. Most of these foods comprise sugar-sweetened beverages, sweets snack bars, chocolates and potato chips, these excessive sugar and salt in these foods impact on children’s health

The National School Nutrition Program (NSNP) currently provides meals to over 9 million primary school children in over 20 000 public schools due to the huge inequality and poverty levels in South Africa [11]. Schools that are selected to receive the NSNP fall under quintile 1 to quintile 2 levels. The quintile system is a reflection of the socio-economic level of the community, with quintile one schools in the poorest communities, while quintile five schools, being situated in high socio-economic communities. According to the South African National Health and Nutrition Examination Survey (2013), it was found that more than half (51%) of children aged 10–14 years take tuckshop money instead of a lunch box to school. Secondly, out of the 51,3% of children who took money to school, nearly half of them spent money on snacks on a daily basis [12]. This was independent of whether they attended a school that was part of the NSNP.

In recent years, dramatic changes in lifestyles and the environment have also brought about significant alterations in children’s eating patterns and food choices. In addition, many eating encounters occur in places outside of the home, such as in schools and surrounding vendors in the community [13]. Therefore, understanding the impact of these food exposures is pivotal if we are to help children establish healthy eating patterns, which contribute to the prevention and delay of non-communicable diseases, such as dental caries, obesity, and diabetes in later life.

This study therefore sought to assess the dietary habits and oral health experiences of primary school learners who attend public schools in Johannesburg. It examined the oral health experiences of the learners, and compared the sugar exposure between children attending NSNP and Non NSNP schools. Additionally, it sought to analyse the relationships between sugar content, oral health experience and body mass index (BMI).

## Methodology

This was a cross-sectional analytical study. The study population included grade three learners, aged 9–11 years in public schools that were located in the Johannesburg Metro District of Gauteng province, South Africa. The focus was on one of the five Johannesburg Metro sub districts, named Johannesburg Central. This sub-district has 134 public primary schools with a total study population of 13928 grade three learners. For a population size of 13928 at an expected frequency of 50% dental caries, with a design effect of 1, a sample size of 235 learners was required at a 95%CI (Epi Info, version 7.2.01).

For inclusion into this study, learners had to have been at the school over a period of at least 2 years as dental caries progresses over 2 years [2]. Learners who had disabilities were excluded. The measurement tools included an observational checklist, a questionnaire, an oral health examination and anthropometric measures for assessing the Body Mass Index (BMI). Recruitment of the participants occurred between the 14/09/2021 to the 27/11/2021.

### Observational checklist

A checklist guided by a published study Halloran et al, 2018) was developed and used to assess the school food environment. It collected information on type of school, type of drinks consumed, and food and snacks the school served and sold. In addition, the availability of vending machines and tuck shops was investigated.

### Questionnaire

The questionnaire used in this study was adapted from the STATS –SA National School Survey and Household income and Red Cap school food environment survey [14]. A sugar intake score (SIS) based on items consumed on the day of the visit and was calculated for NSNP and Non NSNP schools [15]. Estimates of sugar consumption were made based on the following: -

- For daily meals served at school, information on the actual sugar content was obtained from a South African calorie counter online website/ app (fatsecret.co.za).
- For lunch box content and snacks such as chocolates and chips, a sample of the product was obtained and the information about sugar content from the packaging was taken.
- For socioeconomic status, a question was asked on the employment status of at least one parent or caregiver in a home.

### Clinical exam

For the oral health examination, five examiners were trained and calibrated until a kappa score of 80% agreement was reached. Clinical data for dental caries was collected using the dmft/ DMFT index. In addition, a simplified gingival index was used to measure the severity of gingivitis of the learners and was classified according the to the [16]. criteria on a scale ranging from 0.1 to 3.0 (0.1–1.0): mild gingivitis, (1.1–2.0): moderate gingivitis, and (2.1–3.0): severe gingivitis. All examinations were undertaken using mouth mirrors and blunt probes under artificial light in a mobile dental unit as recommended by the World Health Organization [17].

### Anthropometric measures

Anthropometric measures of height and weight were recorded using a digital weight and height scale, a BMI measure was calculated based on the formula, BMI = Weight (kg)/Height (m2) [2]. The weight and height were converted into z scores by age and sex of the learner according to the South African Road to Health birth chart (RTHBC). The z scores were stratified into BMI categories of underweight, normal weight, overweight and obesity according to the percentile weight and height related to the sex and age in the South African RTHC that monitors children’s growth and development patterns [18]. For the purposes of this study, healthy BMI was considered as between 13,7–17,4 as in children BMI is age dependent, therefore a score below or above this range was either underweight or overweight.

### Ethical considerations

An approval from the Wits Human Research Ethics Committee at the Faculty of Health Sciences (M201120) was obtained in order to proceed with the study as well as a written approval from Department of Education and principals of school to gain entry into the schools. Prior to administering the questionnaire and screening the learners, a written informed consent from their parents or guardian was requested in order to enable the learners to participate in the study. Lastly, the learners were also required to provide a written assent before being examined.

### Data analysis

Descriptive statistics such as mean (SD), median (IQR) for continuous variables, in addition frequency and proportions for categorical variables were used to describe the study population. An independent t-test was used to determine if there was a difference in sugar intake and calories intake schools between a NSNP and a Non NSNP school. A Pearson’s pairwise correlation was used to determine the correlations between sugar intake, calories intake, dmft, DMFT and gingiva. In addition, regression analysis was conducted to investigate the relationship between the explanatory variables such as brushing, sugar, calorie intake, money spent on snacks and oral health.

## Results

### Sociodemographic information

Table 1 provides a summary of the socio-demographic profile of the children (N = 107). The mean age of the children was 8.69 years old (SD:0.79) with a BMI of 18.2. The total calorie intake of the children was 643 cal. Majority of the children were females (54.2%), and of the 107 learners, 68.2% were from a NSNP school. Most children possessed a toothbrush and toothpaste (92.5%), brushed at least twice a day (66.40%) and carried lunch and money to school (70.1%; 90.7% respectively). The children from the Non-NSNP school carried more tuck shop money (20.00ZAR) than those from the NSNP school (5.00ZAR). In addition, all the Non NSNP children possessed toothbrushes and paste vs the NSNP school. More (79.4%) children from the Non-NSNP school brushed twice a day. All the children at the Non NSNP school carry lunch to school and 89% of the children at NSNP school carry money for lunch.

**Table 1:** Socio-demographic characteristics of the learners by type of schools (N = 107).

Variables	NSNP school	Non NSNP school	Total
**Continuous variables**	**mean (SD)**	**mean (SD)**	**mean (SD)**
Age	8.67 (0.88)	8.74 (0.57)	8.69 (0.79)
BMI	17.51 (3.10)	19.64 (4.14)	18.19 (3.59)
**Continuous variables**	**Median (IQR)**	**Median (IQR)**	**Median (IQR)**
Amount of spending money (ZAR)	5.00 (3.00-6.00)	20.00 (10.00-20.00)	5 [5–10]
**Categorical variables**	**n (%)**	**n (%)**	**n (%)**
Gender			
Males	34 (46.6%)	15 (44.1%)	49 (45.8%)
Females	39 (53.4%)	19 (55.9%)	58 (54.2%)
Have toothbrush and toothpaste			
No	8 (11.0%)	0 (0.0%)	8 (7.5%)
Yes	65 (89.0%)	34 (100.0%)	99 (92.5%)
Brush how many times a day			
Once	24 (32.9%)	6 (17.6%)	30 (28.0%)
Twice	44 (60.3%)	27 (79.4%)	71 (66.4%)
Thrice	5 (6.8%)	1 (2.9%)	6 (5.6%)
Carries lunch to school			
No	32 (43.8%)	0 (0.0%)	32 (29.9%)
Yes	41 (56.2%)	34 (100.0%)	75 (70.1%)
Carries money to school			
No	8 (11.0%)	2 (5.9%)	10 (9.3%)
Yes	65 (89.0%)	32 (94.1%)	97 (90.7%)

**Fig 1:**
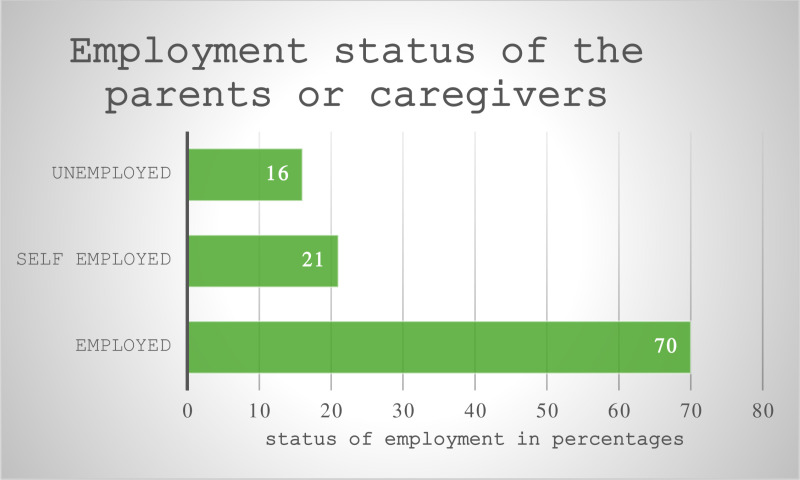
Summary of employment status of parents or caregivers of the children (n = 107). **Footnote: Employed caregivers included, domestic workers, waiters, security guards, retail workers, teachers and policemen.Self-employed refered to caregivers that had local small businesses such as vendor’s, taxi drivers, car mechanics and sales people. Unemployed status referred to nobody working in the household or caregivers being on government pension or social grants.

Fig 1 demonstrate that participants were largely from working class communities as out of the 107 participants, (n = 70;65,4%) of the children’s had care givers that were employed and the unemployment rate was (n = 16;14.9%). The self-employment rate was (n = 21;19.6%).

Table 2 provides a summary of the socio-economic information of the children from the two schools. Caregivers from the Non-NSNP school had proportionally higher levels of employment (82.3% vs 57.5%) and less unemployment (2.9% vs 20.5%) than those from the NSNP school

**Table 2:** Household socio-economic status of the children at the schools.

Employment status	NSNP school N (%)	Non NSNP school N (%)
Employed	42 (57.5%)	28 (82.3%)
Self employed	16 (21.9%)	5 (14.7%)
Unemployed	15 (20.5%)	1 (2.9%)
Total population	73	34

## Oral health experiences

The children from the NSNP school had higher levels of dmft, DMFT and gingival scores respectively [3.14 (3.3); 1.92 (2.2); 0.72 (0.7)]. Among the two schools, majority of the children (44.9%) had normal gingival scores and this was followed by mild gingival scores (36.4%) which was contributed largely by children (45.2%) from the NSNP school (Table 3).

**Table 3:** Oral health experiences of the children in the two schools.

Variables	NSNP school	Non NSNP school	Total
**Continuous variables**	**mean (SD)**	**mean (SD)**	**mean (SD)**
dmft	3.14 (3.39)	1.35 (2.85)	2.57 (3.32)
DMFT	1.92 (2.24)	0.59 (1.41)	1.49 (2.10)
GI score	0.72 (0.77)	0.13 (0.44)	0.53 (0.73)
**Categorical variables**	**n (%)**	**n (%)**	**n (%)**
Gingival scores			
Normal	21 (28.8%)	27 (79.4%)	48 (44.9%)
Mild	33 (45.2%)	6 (17.6%)	39 (36.4%)
Moderate	13 (17.8%)	0 (0.0%)	13 (12.1%)
Severe	6 (8.2%)	1 (2.9%)	7 (6.5%)

### Sugar and calorie exposures

Table 4 Indicates a statically significant difference in the sugar and calorie intake among children that attended a NSNP versus non NSNP school (p < 0.001;p = 0.008), the Non NSNP school had a higher intake of both.

**Table 4:** Sugar and calorie intake between NSNP school and Non NSNP schools.

Domains	NSNP School	Non NSNP School	p-value
Total sugar intake	14.236g (18.783)	45.765g (34.897)	P < 0.001**
Total calories intake	594.536 cal (464.977)	849.441 cal (433.374)	P = 0.008 **

Table 5 Results indicated a negative correlation between calorie intake, sugar intake and DMFT, GI score. As calorie intake increases, DMFT decreased (p = 0.015) and as sugar intake increased the GI score decreased (p = 0.018).

**Table 5:** Correlation between sugar and calorie intake with DMFT/dmft and GI score.

	Total sugar intake		Total Calories intake	
	Correlation coefficient	p-value	Correlation coefficient	p-value
**DMFT**	-0.1635	0.0924	-0.2343	0.0151**
**dmft**	-1.1008	0.3017	-0.0278	0.7761
**GI score**	-0.2273	0.0185**	-0.1667	0.0861

Table 6 provides a summary estimate of the daily sugar intake at both schools and indicate that the food from school meals at the NSNP school provide less sugar intake [mean 11.65 (9.6)] than the combined mean amount [24.53 (24.54)] from the purchased snacks and lunchboxes all the children in the two schools are exposed to.

**Table 6:** Estimate of sugar content of food consumed at both schools per day.

Variable	NSNP school N(95%CI) Mean(SD)	Non-NSNP school	Total
Sugar content of meals served by school (g)/ per day	477.78 (353.52- 602.04) 11.65 (9.6)	--------	477.78 (353.52- 602.04) 11.65 (9.6)
Sugar content from purchased snacks and lunchboxes (g)/ per day	819.13 (647.05-1135.21) 17.14 (16.86)	1218.47 (863.71-1573.24) 35.84 (29.91)	2109.6 (1657.07-2562.13) 52.98 (24.54)
p-value			0.0001**

### Relationship between oral health experiences and other variables

Table 7 provides a summary on the relationship between dmft and other variables such as access to toothbrush and paste, brushing frequency, BMI, sugar and calorie intake and spending money on the dmft in children. The adjusted multivariate linear regression results suggests that none of the variables investigated affected the dmft scores.

**Table 7:** Relationship between variables and (dmft).

dmft	Univariate regression		Multivariate linear regression	
	Unadjusted Coefficient (95%CI)	p-value	Adjusted Coefficient (95%CI)	p-value
**Toothbrush and paste**				
No	0		0	
Yes	-1.545 (-3.960 to 0.869)	0.207	-1.55 (-4.16 to 1.07)	0.243
**How often they brush**				
Once	0		0	
Twice	-1.495 (-2.886 to -0.103)	0.036	-0.95 (-2.41 to 0.52)	0.202
Thrice	1.700 (-1.158 to 4.558)	0.241	1.92 (-0.85 to 4.70)	0.173
**BMI**	-0.078 (-0.256 to 0.101)	0.390	-0.05 (-0.22 to 0.13)	0.618
**Sugar intake**	-0.012 (-0.034 to 0.011)	0.302	-0.01 (-0.03 to 0.02)	0.192
**Calories intake**	-0.0002 (-0.002 to 0.001)	0.776	-0.0001 (-0.002 to 0.001)	0.919
**Amount of spending money**	-0.025 (-0.059 to 0.008)	0.136	-0.02 (-0.06 to 0.12)	0.267

For the relationship between DMFT and similar variables, Table 8 indicates that brushing with a toothbrush and toothpaste had a significant impact on the DMFT (p = 0.047).

**Table 8:** Relationship between variables and oral health (DMFT).

DMFT	Univariate regression		Multivariate linear regression	
	Unadjusted Coefficient (95%CI)	p-value	Adjusted Coefficient (95%CI)	p-value
**Toothbrush and paste**				
No	0		0	
Yes	-1.356 (-2.870 to 0.158)	0.079	-1.55 (-3.07 to -0.02)	0.047**
**How often they brush**				
Once	0		0	
Twice	-0.263 (-1.176 to 0.649)	0.568	-0.02 (-0.83 to 0.79)	0.960
Thrice	-0.533 (-2.408 to 1.341)	0.574	-0.44 (-1.97 to 1.10)	0.574
**BMI**	0.024 (-0.089 to 0.138)	0.669	0.01 (-0.09 to 0.11)	0.795
**Sugar intake**	-0.011 (-0.026 to 0.002)	0.092	-0.002 (-0.02 to 0.01)	0.739
**Calories intake**	-0.001 (-0.002 to -0.0002)	0.015	0.001 (0.002 to 0.0002)	0.118
**Amount of spending money**	0.003 (-0.016 to 0.022)	0.729	0.01 (-0.01 to 0.03)	0.436

In terms of relationship between gingival scores and the variables, the adjusted regression analysis found that high BMI (OR: 0.85; p = 0.019) sugar intake (OR: 0.99; p = 0.041) was associated with the odds of reduced gingival scores, see Table 9.

**Table 9:** Relationship between variables and gingival scores.

Gingiva (GI)	Univariate regression		Multivariable ordinal regression	
	Unadjusted Odds ratio (95%CI)	p-value	Adjusted Odds ratio (95%CI)	p-value
**Toothbrush and paste**				
No	1		1	
Yes	0.497 (0.138 to 1.791)	0.285	0.377 (0.064 to 2.229)	0.282
**How often they brush**				
Once	1		1	
Twice	0.516 (0.233 to 1.140)	0.102	0.638 (0.257 to 1.581)	0.331
Thrice	1.245 (0.198 to 7.835)	0.815	1.073 (0.140 to 8.23)	0.946
**BMI**	0.822 (0.723 to 0.936)	0.003	0.848 (0.738 to 0.938)	0.019**
**Sugar intake**	0.973 (0.956 to 0.991)	0.003	0.998 (0.957 to 0.999)	0.041**
**Calories intake**	0.999 (0.998 to 1.000)	0.039	1.000 (0.998 to 1.001)	0.368
**Amount of spending money**	0.980 (0.957 to 1.004)	0.104	0.993 (0.968 to 1.020)	0.610

## Discussion

Of the 107 eligible learners, 68% were from a school with a feeding scheme and 31.8% with no feeding scheme. The mean (SD) of BMI, dmft, and DMFT were 18.19 (3.59), 3.14 (3.39), and 1.49 (2.10), respectively. The learners in this cohort were marginally overweight and had higher caries experience (dmft -1,2: SD:2.3); (DMFT-0.90:SD:1.7) than children of similar schools in the nearby Tshwane District [19]. The total sugar consumed by learners from Non-NSNP schools was significantly higher (p < 0.001). The Pearson’s correlation results revealed a negative correlation between calorie and sugar intake and DMFT and Gingival scores. Furthermore, upon undertaking regression analysis and adjusting for confounders, possessing a toothbrush and toothpaste was protective for the DMFT and a decrease in the gingival score was associated with a high BMI and sugar intake at both schools.

In as much as the child population came from a working-class community evidenced by (65.4%) of the caregivers being employed. The economic status of children from the Non-NSNP school was higher than that of the NSNP school as reflected in their proportionally higher employment rate (82.3% vs 57.5%). In addition, the Non-NSNP children had full access to oral cleaning aids and more (20.00 ZAR) pocket money for purchasing school lunch. Hence, they were not beneficiaries of the food program and did not fall under quintile 1 and 2 schools that were classified as poor. An interesting phenomenon in the study findings was that although the children from the Non NSNP school had higher sugar and calorie intake, their disease burden was less, their DMFT, dmft and gingival scores were low. This protective association of a higher socioeconomic status against dental caries was demonstrated by a systematic review exploring the prevalence and associated factors of dental caries among children in Low-Middle income countries [20]. In the review it was found that children from mothers who had secondary education or more had lesser odds of being affected by dental caries (OR=0.96;95%CI = 0.64 to 1.44). In addition, children belonging to the middle socio-economic status had a 20% less chance of being affected, whereas children from a low socioeconomic background had a 52% chance of acquiring dental caries [20]. These results are reflective of what we are witnessing in our population.

In the study, access to toothbrushes and paste was found to be protective against dental caries in permanent teeth. However, it was still concerning that 7.5% of the children did not possess a toothbrush. Recent studies demonstrated that although majority of the children from Low Middle Income (LMIC) countries may have access to a toothbrush, access to fluoridated toothpaste continues to be a challenge. In Eritrea, a study done among 12–15 year olds demonstrated that although 97% of the children possessed toothbrushes, 89% were unaware of whether the toothpastes used had fluoride or not [21]. Furthermore in Malawi, among 95% of 11–14 year olds that had toothbrushes, agents used on the toothbrushes other than toothpaste included ashes and salt [22]. Therefore, poor access to fluoridated toothpaste coupled with poor oral hygiene practices, a diet high in sugar consumption and low levels of oral health literacy among caregivers accelerates caries development and contributes to poor oral health [20,23].

The study results significantly highlight that the mean sugar content of the food served at NSNP schools is much less than the sugar consumed from lunch boxes and purchases at both the schools (see Table 6), demonstrating the benefits of regulated school meals. Evidence from literature indicate a similar phenomenon of higher level of sugar content being found in packed school lunches in comparison to served school meals in the UK [24,25]. Of concern is that those on school meals consuming on average (11,6g) per day and additionally purchasing school snacks that add (17.1g), potentially consume 28.7 g of free sugars per day thus exposing themselves to sugar above the recommended daily limit of 25g proposed by the WHO & American Heart Foundation [26,27]. This demonstrates that all our participants had easy access to unhealthy snacks in the school food environment irrespective of NSNP and non NSNP.

The NSNP and non NSNP schools appeared not to have a health promoting school food environment and nutrition was not controlled. The published literature cites reasons linked to the challenge, these includes poor food choices made by parents who pack lunchboxes or give money to learners for purchasing snacks [12]. Secondly, children often prefer unhealthy snacks and furthermore, school vendors, particularly in South Africa are reported to claim to have no facilities in storing healthy perishable fruit and vegetables for sale [12]. Ventura et al, 2011 also suggest that children have learned food preferences for sweet taste from an infant age, therefore when the home environment does not assist the child in changing those taste preferences by repeatedly exposing them to unhealthy foods, the affinity for sugar preference continues and subsequently influences food choices at school [28]. Hence a relationship exists between sugar consumption at home and sugar consumption choices at schools. Such a relationship should be considered when planning preventative interventions [29].

As our study considered a healthy BMI appropriate for the children’s age to be between (13.7–17.4), the participants were slightly overweight with a mean BMI score of 18.2. There was a significant relationship between low gingival score with BMI and sugar, showing that the odds of a reduced gingival score were linked with increasing BMI and sugar. This may have been attributed to that the majority of our participating children had normal to mild gingival scores, they had access to toothbrush, paste (92.5%) and brushed twice a day (66.4%) which is protective against the sugar and calorie exposures [20]. There was however no statistically significant relationship between dental caries, BMI and sugar. This was in contrast with some study findings. In Indonesia it was found that a low BMI was associated with a high DMFT as a result of the pain experienced when they had to be chewing food and eating adequately (Wening et al, 2019). In turn among children (n = 169) in Pretoria, South Africa, it was found that the BMI score was inversely proportional to the number of decayed teeth, therefore a high BMI was linked with low scores of dental caries (r = -0.187, p = 0.042). Although the BMI was not associated with dental caries in our study, it was associated with low gingival scores, inferring that the socio-economic status of the children provided them an environment for positive oral hygiene behaviour resulting in low gingival inflammation. [30].

## Limitations of the study

This was an observational study, and we were not able to control for learners who brought lunchboxes at schools and effect of exercise on BMI. However, we were able to adjust for confounders using the multivariate regression analysis. We experienced a low response rate from the target sample size of (N = 235) as data had to be collected during the aftermath of the COVID pandemic where schools were allowing access under stringent infection control protocols and thus some parents were reluctant for their children to participate in the study. The limited sample size in the NSNP and Non NSNP schools limited us from making extensive analytical comparisons, but the information gathered provided insight of the level of sugar exposures at school settings. In addition, although the BMI and sugar intake reduced the odds of a high gingival score, the effect size was small thus further research with a larger sample needs to be undertaken to assess the real effect difference.

## Conclusion

Our study revealed that the school food environment at both NSNP and non NSNP schools had poor controls over sugar consumption and purchasing behaviour of the learners. There was a relationship between the BMI, sugar intake and gingival scores which could be attributed to socioeconomic factors, access to food, toothbrush and paste among the group of participants. Given the high levels of sugar exposure among the participants at schools, it is therefore recommended that in addition to the standard meals served, schools develop and adopt policies on school dietary consumption as there is evidence that such policies potentially improve dietary behaviours of children [31].

## Supporting information

S1 Table Copy of Dataset cleaned. (XLS)
